# Capturing quality of education in cognitive assessments in diverse populations

**DOI:** 10.1002/alz70857_096676

**Published:** 2025-12-24

**Authors:** Renelle Bourdage, Eithne Macdonald, Souhaila Tamali, Sandjai Ramsaran, Clara Calia, Sanne Franzen

**Affiliations:** ^1^ Erasmus University Medical Center, Rotterdam, South Holland, Netherlands; ^2^ Laboratoire Mémoire Cerveau et Cognition (UR 7536), Université Paris Cité, Boulogne Billancourt, France; ^3^ School of Health in Social Science, The University of Edinburgh, Edinburgh, United Kingdom; ^4^ Erasmus School of Social and Behavioral Sciences, Erasmus University Rotterdam, Rotterdam, Netherlands; ^5^ Faculty of Behavioral and Movement Sciences, Vrije Universiteit Amsterdam, Amsterdam, Netherlands; ^6^ School of Health in Social Science, University of Edinburgh, Edinburgh, United Kingdom; ^7^ Erasmus MC ‐ University Medical Center, Rotterdam, South Holland, Netherlands

## Abstract

**Background:**

Education and literacy are important cultural variables in neuropsychological assessments, yet education is often measured by years of education or level, which fails to reflect education quality. Literacy is often assessed with reading level or vocabulary, which is typically not possible in immigrant groups. Factors such as access to resources and teacher‐student ratio influence education quality and literacy, making comparisons across cultures difficult and inconsistent. This study developed a mixed‐methods Quality of Education (QoE) Interview and collected data from older, culturally and educationally diverse participants to identify key components to best systematically measure quality of education.

**Method:**

Healthy controls (aged 50 years^+^) originally from Morocco and Suriname who moved to the Netherlands, were included in the study. The QoE Interview is composed of 12 closed answer questions, where participants are encouraged to reflect and elaborate if possible (e.g., Did you have access to textbooks?), and three open‐ended questions. The open‐ended questions ask participants what they believe are signs of a ‘good’ or ‘bad’ primary school, and about their experience answering the interview questions.

**Result:**

The QoE Interview outlined important dissimilarities in school experience mediated by socio‐economic status. Some participants stated that they were poor and therefore did not (or not often) have access to school books, materials or school library. Other participants did not attend school because only boys were permitted to go. For the open‐ended question on signs of a “good” school, frequent responses were good teachers (e.g., supportive), and access to resources and spacious classrooms. For “bad” schools, participants cited poor furnishings, small classrooms, unsupportive teachers, unsanitary facilities, and student conflicts. All participants found the QoE Interview had good questions, though, remarkably, several participants noted some questions were emotionally challenging as they highlighted socio‐economic disparities.

**Conclusion:**

The QoE Interview seems to capture quality of education and may serve as a guide to a shortened standardize measure of quality of education for future research, such as studies examining dementia risk or longitudinal studies of cognitive decline. Studies are in progress to cross‐validate the tool with other quality of education indicators.

